# The Dental Profession in France

**Published:** 1875-08

**Authors:** Mordaunt Stevens


### ARTICLE VII.

### *The Dental Profession in France.*

BY MORDAUNT STEVENS, M. R. C. S. E., L. D. S., D. D. S., &C.

Two months ago at the request of some of our readers we announced that in all probability a meeting of dentists would take place in Paris towards the end of this month to discuss the clauses of a new law to regulate the practice of dentistry in France. We made this announcement under protest, assuring the gentlemen who had come to Paris to discuss this matter with us, that, to say the least, such a notion was premature, but our confreres, more sanguine even than ourselves argued that surely in Paris (a city in which dentists postively swarm,) a certain number of practitioners could be found ready to take the initiative, and to meet to consider what had best be done for the general good of the profession. The event has proved, alas, that they were premature in their anticipations, and that we were unfortunately right. Our provincial readers cannot form an idea of the difficulty there is in bringing about this long talked of dental congress; there are so many small coteries and cliques, so much petty jealousy, rivalry, and competition, in fact, such a total absence of *esprit de corps* and true professional feeling, that the task of bringing so many strange sheep into one fold may fairly be looked upon as well nigh hopeless for the present. On the one side we have the doctors of medicine, graduates of French universities; these gentlemen, looking upon dentistry as a branch of surgery, consider, with justice, that none but qualified medical men have the right of practising the speciality. The law is against them it is true, they submit, but they look upon old dentists practising *sine* diplmoa, and upon all foreigners, as interlopers, in fact as so many poaches intruding on their estate. On the other side we have dentists who have duly served their apprenticeship and are thoroughly competent in the more mechanical departments of the profession; they look on the question in quite another light. "While the doctors," say they, "were studying anatomy and surgery, we were toiling in the workroom, and acquiring a more practical knowledge of our profession, and we consider that we ought in no way to be eclipsed by our more scientific brethren." The foreign dentists claim that they have raised dentistry in France to the position it now occupies; they are perhaps a little proud of the special training they have received in English or American dental colleges, and appear not to be so willing to hold out the hand of good-fellowship to those who have been brought up under the old system as they ought to be. We must not forget the charlatans; they reap a golden harvest from the present state of things, and naturally will do all in their power to prevent any legislative reform.

Our worthy "confreres" do not disagree merely on what may be called public questions, private dissensions exist between them, and it is hard indeed to find two dentists who are on speaking terms with one another. We are now talking of the respectable portion of the craft, but even the charlatans are divided amongst themselves; these worthies, the very pariahs of our profession, will not even herd together; they hate their equals even more than they hate their superiors. We hope our readers will not accuse us of exaggerating the state of affairs. We have considered the question calmly and we trust dispassionately. Here in the metropolis which boasts of ruling the universe on all questions appertaining to science and art; in the town where almost every door is adorned with the brass plate of one of our craft, in a city which has been called a hive of dentists, we cannot find twenty amongst the many good men and true who practice here, ready to forget private squabbles and do something for the general good. No! Some are indifferent, others looking upon themselves as *the only bright shining lights*, will not associate with their fellows; others again, have some grievance against one or more of their brethren and will not join any society for fear of having to meet those who have incurred their displeasure. The story of the mote and the beam over again; they forget that some of their own bitter sayings against their neighbors may have been reported and magnified. Here let us stop; we have purposely avoided discussing the numerous letters we have received on the subject of the congress; as we do not care to enter, unless it be forced upon us, into a paper warfare. We have depicted the state of the profession in France as it is at present; it is not our fault if the picture be not attractive.—*London Review of Dental Surgery.*

